# The impact of COVID-19 on cultural tourism: art, culture and communication in four regional sites of Queensland, Australia

**DOI:** 10.1177/1329878X20952529

**Published:** 2021-02

**Authors:** Terry Flew, Katherine Kirkwood

**Affiliations:** Queensland University of Technology (QUT), Australia

**Keywords:** Cairns, COVID-19, creative industries, cultural tourism, Gold Coast, Indigenous, Outback, regional Queensland, Sunshine Coast, the arts

## Abstract

The arts, cultural and creative industries are among the most adversely affected sectors of the economy in the wake of COVID-19 social distancing measures, travel restrictions and prohibition of large gatherings of people. Focusing on Cairns, the Gold Coast, Central West and the Sunshine Coast – four regional areas of Queensland, Australia – this article provides an overview of impacts on cultural tourism and considers the prospects for regional cultural tourism as part of a ‘creative economy’ revival.

## COVID-19 and its cultural impacts

In Australia, the arts, cultural and creative industries have been among those sectors of the economy most seriously impacted by COVID-19, with responses at various levels of government focused upon social distancing, travel restrictions, prohibitions on gatherings of large groups and the resulting economic recession. Deloitte Access Economics estimated the cumulative impact on wages and profits of COVID-19 on arts and recreation to be AU$6 billion, making it the second hardest-hit sector after accommodation and food services (Richardson, 2020). The Australian Bureau of Statistics (ABS) found that only 47% of businesses in the arts and recreation services sector were trading in the week commencing 30 March 2020, making it the sector hardest hit by COVID-19-related business shutdowns in Australia. The ABS also found that 94% of businesses classified as being in Arts and Recreational Services had been adversely affected by government restrictions arising from COVID-19, as compared to 53% of businesses as a whole (Australian Bureau of Statistics, 2020).^1^

While the impact on arts and cultural sectors has been very adverse generally, some interesting questions arise out of a potential shift from international to domestic tourism. For localism, arts and culture particularly intersect around the area of cultural tourism. The United Nations World Tourism Organization (UNWTO, 2018) define cultural tourism as ‘a type of tourism activity in which the visitor’s essential motivation is to learn, discover, experience and consume the tangible and intangible cultural attractions/products in a tourism destination’. As the cultural tourism phenomenon accelerated in the 1990s, distinct sub-sectors emerged, including ‘heritage tourism, arts tourism, gastronomic tourism, film tourism and creative tourism’ (Richards, 2018: 12). In Australia, an exemplar of the relationship between the arts and cultural tourism has been the Museum of Old and New Art (MONA), the private museum established by entrepreneur and arts *animateur* David Walsh in Hobart in 2011. The ‘MONA Effect’ refers to the growth of visitors to Tasmania, and particularly to Hobart, after the MONA opened in 2011. With events such as MONA FOMA in summer and Dark MOFO in winter, MONA has come to exemplify cultural tourism’s power as an economic and social attractor, achieving what were once seen as impossible targets of bringing over 100,000 people to Hobart in the middle of winter (Franklin, 2014).

Prior to COVID-19, Richards (2018: 12) noted that ‘over tourism’ in some parts of the world – particularly World Heritage Sites – was becoming problematic. Now, as populations and policy-makers attempt to simultaneously prop up public health and the economy, the challenge for cultural tourism operators will be remaining solvent during a crisis that restricts the movement of people. Looking at Queensland, Australia, the cessation of international leisure travel has hit traditional tourist hubs like the Gold Coast and Far North Queensland particularly hard. But early signs are that there may be a renewal of domestic tourism, particularly in places that are readily accessible by car and within state boundaries. This short article considers the prospects for regional renewal based around cultural tourism in four regional areas of Queensland: Cairns, the Gold Coast, Central West and the Sunshine Coast. It also briefly notes the impact of other enabling or inhibiting factors, most notably cultural policy settings and the impact of COVID-19 on locally based media.

## COVID-19 and cultural tourism in four Queensland regions

As one of the Australian states most economically reliant upon tourism, Queensland has sought to tap into cultural tourism’s potential, particularly around Indigenous arts and culture (Langton, 2018).^2^ But COVID-19 has significantly curtailed both tourism and arts and culture, with restrictions upon travel and movement, out-of-home activities, and large gatherings. Across the four Queensland regions of Cairns, the Gold Coast, the Sunshine Coast and the outback Central West region, COVID-19 presents a major test of resilience of the cultural and creative sectors, as international and interstate travel stopped in March 2020, and Queenslanders were largely quarantined to their local areas. State and Federal governments have been particularly focused upon developing rescue packages for Cairns and the Gold Coast in the wake of COVID-19 (Department of Infrastructure, Transport, Regional Development and Communications, 2020), but the pandemic’s onset has given sharp focus to the question of the ability of local cultural activities and infrastructure to appeal to local populations. Even as travel bans are gradually lifted at a national level, a focus on attracting local visitors is likely to remain for some time.

Of the four regions, Cairns has done the most to diversify tourism to appeal to its local catchment. Mayor of the Cairns Regional Council, Cr. Bob Manning OAM, identified the region’s ‘three great tourism assets as the reef, the rainforest, and local Indigenous culture’ (Cunningham et al., 2019: 2), and it is the latter that has the most scope to build local support, as assessments of the impact of COVID-19 in the region point to the loss of AU$2.5 billion in visitor expenditure and 13,650 jobs, or 9% of all jobs in the region (Department of Infrastructure, Transport, Regional Development and Communications, 2020). Cairns Regional Council’s (2017) *Strategy for Culture and the Arts 2022* focused on aligning cultural policy more directly to supporting tourism and cultural tourism, developing a unique point of difference through supporting and celebrating Aboriginal and Torres Strait Islander culture, expanding engagement from a local audience to a broader footprint and building place identity and the creative economy. In this way, it was proposed that Cairns be promoted as an ‘international creative powerhouse’, strengthening the region’s reputation as a marketplace for Aboriginal and Torres Strait Islander cultural and creative expression, and advocating for and showcasing local creative talent. Although COVID-19 constrains the strategy’s international angle, the Cairns Regional Council’s efforts to preserve local heritage and promote local artists, historical museums and societies serve to enhance the region’s reputation as a cultural tourism destination.

The Gold Coast has also been particularly hindered by COVID-19. While exact assessments of impact are not currently available, one indicator is that hotel occupancy rates fell from 84% in February 2020 to 4% by April 2020 (Huxley, 2020). The Gold Coast is particularly impacted by the absence of international flights, theme park closures, the pause on film and television production, and the cancellation of conventions and events. For Village Roadshow’s theme parks, closures impact 4000 employees and stifle the generation of AU$50 million in construction work these sites bring to the region each year. The Gold Coast has taken major steps to develop its cultural infrastructure over the last decade, including construction of the Home of the Arts centre in Southport, a music industry strategy, and Indigenous cultural tourism sites such as the Jellurgal Aboriginal Cultural Centre and the Yugambeh Museum. The appeal of these local cultural sites to the community, and travellers in the Gold Coast’s immediate vicinity, are vital for the future of tourism in one of Australia’s major destinations, particularly if restrictions on theme park activity continue.

The Central West region has been a largely unheralded tourism success story in Queensland with landmarks including the Qantas Founders Museum (aviation history and science, Longreach), the Stockman’s Hall of Fame (agricultural heritage, Longreach), the Age of Dinosaurs Museum (science and paleo-tourism, Winton), the Dinosaur Dreaming Trail (Indigenous history and paleo-tourism), the Waltzing Matilda Centre (cultural history, Winton), the Royal Open Air Theatre (entertainment heritage, Winton) and the Tree of Knowledge (political history, Barcaldine), as well as established annual festivals such as the Vision Splendid Outback Film Festival and the Way Out West Music Festival. It is possible that these attractions will do well post-COVID-19, as many domestic travellers forego air travel, and drive to locations where they can engage with local history and storytelling.

As Queensland’s state government worked to implement and then ease restrictions, outback areas were at times afforded less invasive restrictions or speedier re-openings of business (Queensland Government, 2020b). From a cultural tourism perspective, this makes Central West attractions appealing destinations, particularly when sites in more densely populated areas of Southeast Queensland remained bound by tighter restrictions. Many of the aforementioned Central West attractions are in the Local Government Areas of Winton Shire Council, Barcaldine Regional Council and Blackall-Tambo Regional Council. These are noted in the Queensland Government’s (2020a) *Roadmap to easing Queensland*’*s restrictions* as outback areas.

Of the four regions considered, the Sunshine Coast has made the fewest investments in linking cultural tourism to a wider creative economy strategy, although it developed its first Arts Plan in 2018 (Sunshine Coast Council, 2018). Its focus has been more upon developing IT-related start-ups, with tourism being more linked to lifestyle and regional amenity, but new business development initiatives such as The Refinery are focused upon arts and cultural practitioners building sustainable businesses. Whether post-COVID-19 opportunities emerge depends in part upon whether policy-makers can extend the Sunshine Coast region’s appeal beyond its natural assets to encompass unique cultural tourism experiences, particularly those that engage with the region’s Indigenous culture and heritage.

We conclude with consideration of two relevant issues. One is the impact of current arts and cultural policy in Australia. Much has been made of the inadequacy of the Federal Government’s response to the impact of COVID-19 on the arts, seen as accentuating already strained relationships between the cultural community and the Government. Shadow Minister for the Arts, Tony Burke (2020) MP, referred to the Morrison Government’s abandonment of the arts and entertainment sector throughout COVID-19, claiming that he was ‘stunned by their apparent willingness to sit by and do nothing while an entire industry crumbled’.

Furthermore, a study by A New Approach found that local government per capita expenditure increased by 11% and State and Territory government expenditure increased by 3.9% between 2007–2008 and 2017–2018, while Federal expenditure fell by 18.9% per capita over the same period. This means that responsibility for cultural expenditure is split more evenly between the three tiers of government than it was a decade ago. In 2017–2018, the Federal government contributed 39% of funding (down from 45.7% in 2007–2008), while State and Territory governments contributed 34.8% (up from 31.9% in 2007–2008) and local governments contributed 26.2% (up from 22.4% in 2007–2008) (A New Approach, 2019: 5). If the future of arts and cultural policy is that it will be increasingly funded by state and local governments, rather than being framed within overarching national cultural policies, then state and local governments in Queensland are well placed to respond to a ‘turn to the regions’ driven by locally based cultural tourism.

One development that will affect all of the regions considered here is COVID-19’s impact upon local media, particularly regional newspaper closures. In late May 2020, News Corporation announced that 100 of its print mastheads would either discontinue production or become digital-only. While the *Cairns Post* and *Gold Coast Bulletin* will continue print production, the *Sunshine Coast Daily* and all papers catering to Central and Western Queensland became digital-only, and may cease production altogether in the near future (Doran, 2020). This constitutes a potentially massive blow to the communications infrastructure that enables cultural development in these communities. It presents a serious obstacle to revitalising regions as cultural centres in their own right, and comes at a time when the post-COVID-19 cultural and tourism environment points towards the importance of embracing localism in ensuring long-term sustainability in regional creative economies.

## Notes

1. This study draws upon the ARC Linkage-Project *Australian cultural and creative activity: A population and hotspot analysis* (LP160101724) led by Queensland University of Technology in partnership with the University of Newcastle, Arts Queensland, Create NSW, Creative Victoria, Arts South Australia and the WA Department of Culture and the Arts.

2. A detailed account of cultural tourism strategies in four Queensland regions (Cairns, Gold Coast, Central West and Sunshine Coast) in the context of their cultural economy strategies can be found in (Cunningham et al., 2020).
